# Pycnogenol® Induces Browning of White Adipose Tissue through the PKA Signaling Pathway in Apolipoprotein E-Deficient Mice

**DOI:** 10.1155/2018/9713259

**Published:** 2018-01-17

**Authors:** Huiying Cong, Wenxia Zhong, Yiying Wang, Shoichiro Ikuyama, Bin Fan, Jianqiu Gu

**Affiliations:** ^1^Department of Endocrinology and Metabolism, The First Hospital of China Medical University, No. 155 Nanjing North Street, Shenyang 110001, China; ^2^The Endocrine Institute and the Liaoning Provincial Key Laboratory of Endocrine Diseases, The First Hospital of China Medical University, No. 155 Nanjing North Street, Shenyang 110001, China; ^3^Department of Clinical Investigation, Department of Diabetes, Endocrine and Rheumatic Diseases, Oita San-ai Medical Center, 1213 Ichi, Oita 870-1151, Japan; ^4^Department of Neurology, Shengjing Hospital, China Medical University, No. 39 Huaxiang Road, Shenyang 110022, China

## Abstract

Beige adipocytes in white adipose tissue (WAT) have received considerable recognition because of their potential protective effect against obesity. Pycnogenol (PYC), extracted from French maritime pine bark, has anti-inflammatory and antioxidant properties and can improve lipid profiles. However, the effect of PYC on obesity has never been explored. In this study, we investigated the effects of PYC on obesity and WAT browning in apolipoprotein E- (ApoE-) deficient mice. The results showed that PYC treatment clearly reversed body weight and the mass of eWAT gain resulting from a high-cholesterol and high-fat diet (HCD), but no difference in food intake. The morphology results showed that the size of the adipocytes in the PYC-treated mice was obviously smaller than that in the HCD-fed mice. Next, we found that PYC upregulated the expression of genes related to lipolysis (ATGL and HSL), while it decreased the mRNA level of PLIN1. PYC significantly increased the expression of UCP1 and other genes related to beige adipogenesis. Additionally, PYC increased the expression of proteins related to the protein kinase A (PKA) signaling pathway. The findings suggested that PYC decreased obesity by promoting lipolysis and WAT browning. Thus, PYC may be a novel therapeutic target for obesity.

## 1. Introduction

The prevalence of obesity, which has increased regardless of region and country in recent decades, is associated with abnormal adipose tissue distribution [1, 2]. Obesity, especially central obesity, is an independent risk factor for the onset of type 2 diabetes. Excessive body fat accumulation has a profound effect on tissue insulin sensitivity, which in turn affects systemic glucose homeostasis; the underlying molecular mechanism is not entirely clear. In mammals, adipose tissue is composed of white adipose tissue (WAT) and brown adipose tissue (BAT), and their dysfunction can lead to overweight and obesity [2]. WAT stores energy in the form of triglycerides, and excess WAT leads to metabolic abnormalities [3]. However, BAT is specialized to burn fat and generate energy in the form of heat because of its higher mitochondrial content with UCP1 expression, which plays an important role in the regulation of energy balance [3, 4]. Recently, a new type of adipocyte, brown-like adipocytes (also called beige adipocytes), was discovered in WAT. Beige adipocytes are also thermogenic adipocytes because of their large mitochondrial content with UCP1 expression, and they generate heat under cold exposure. Beige fat exhibits similar metabolic properties to BAT, and both control energy homeostasis [4–7]. Thus, brown fat and beige fat are potential therapeutic targets for obesity [4, 7].

UCP1, a key regulator of thermogenesis, is regarded as a BAT-specific transporter protein, and its activation gives BAT and beige adipocytes the capability of thermogenesis [4, 8, 9]. It is recognized that the function of UCP1 is activated by the sympathetic nervous system (SNS). The SNS is known to promote lipolysis via the *β*_3_-adrenergic signaling pathway in adipose tissue. *β*_3_-adrenergic receptors (*β*_3_-ARs) are expressed on the surface of adipocytes and activate protein kinase A (PKA), leading to increased lipolysis by activating hormone-sensitive lipase (HSL). PKA further activates p38 mitogen-activated protein kinase (MAPK) and increases the expression of UCP1, thereby promoting thermogenesis in BAT [10, 11]. However, the increase in UCP1 expression manifests as white adipocytes, which induce the browning of WAT [12, 13]. Many transcriptional regulators, such as PPAR*γ*, PGC1*α*, PRDM16, and Cidea, participate in the process of WAT browning and enable WAT to achieve similar biological functions as BAT [12, 13].

Pycnogenol (PYC), one of the natural antioxidants extracted from French maritime pine bark, is composed of procyanidins, phenolic acids, and bioflavonoids [14, 15]. Previous studies have shown that PYC has a beneficial protective effect against cardiovascular disease via its anti-inflammatory effects [16, 17]. Clinical studies have also shown that PYC significantly reduced waist circumference and triglyceride and cholesterol levels [18, 19]. In vitro, PYC supplementation decreased the triglyceride level by stimulating lipolysis in adipocytes [11]. However, few studies have focused on the effects of PYC on the browning of white adipocytes. Apolipoprotein E- (ApoE-) knockout mice are the most widely used atherosclerosis mouse model [20]. We also chose this model to explore the effect of PYC on atherosclerosis in the original study, the results of which have been published [16]. At the same time, we found that ApoE-knockout mice fed with a high-cholesterol and high-fat diet (HCD) developed obesity, which was improved in the PYC-treated groups. In this study, we investigated, for the first time, the effects of PYC on the expression of genes related to the browning of WAT in ApoE-deficient mice fed with an HCD, which may provide a novel strategy for preventing and treating obesity.

## 2. Materials and Methods

### 2.1. Animals and Treatment

The animal experiment was approved by the Animal Research Committee of China Medical University (Shenyang, China). One hundred male ApoE-deficient mice (7 weeks) were obtained from Vital River (Beijing, China), and they had free access to food and water. After adapting to the feeding regimen, the mice were randomly divided into 3 groups, namely, the blank group (*n* = 50), the 30 mg·kg^−1^·day^−1^ PYC plus HCD group (LoPYC + HCD group, *n* = 25), and the 100 mg·kg^−1^·day^−1^ PYC plus HCD group (HiPYC + HCD group, *n* = 25), which were treated via oral gavage for 10 weeks with distilled water, 30 mg·kg^−1^·day^−1^ PYC, and 100 mg·kg^−1^·day^−1^ PYC, respectively. PYC, which was generously provided by Horphag Research (Geneva, Switzerland), was dissolved in 0.1 ml of distilled water and administered orally for 10 weeks. During the first 2 weeks, these mice were fed a regular rodent chow diet. After 2 weeks, the mice in the blank group were divided into 2 subgroups, that is, the normal diet group (ND group, *n* = 25) and the HCD group (*n* = 25), which were fed a regular rodent chow diet and an HCD (Research Diet; NJ, USA), respectively, for 8 weeks. The mice in the PYC-treated groups were switched to an HCD for 8 weeks. Body weight and food intake were measured once per week. At the end of the experiment, all mice were sacrificed, and epididymal fat was harvested and stored at −80°C.

### 2.2. Histology

The epididymal fat samples were cut into sections (10 *μ*m) using a Leica cryostat and were then stained using hematoxylin and eosin (H&E) to visualize the size of adipocytes in epididymal white adipose tissue (eWAT) using a microscope (OLYMPUS BX51, Japan) (*n* = 5 mice per group). In each section from each mouse, the number of cells within four randomly chosen areas (100 × 100 *μ*m) was counted, and the mean value was calculated.

### 2.3. Immunohistochemistry

For UCP1 staining, tissues were embedded in paraffin, and tissue sections were cut to a thickness of 5 *μ*m. Paraffin sections were deparaffinized in xylene, hydrated with ethanol, and subjected to heat-mediated antigen retrieval. Then, the sections were incubated with 3% H_2_O_2_ for 15 minutes and blocked with 1/100 diluted goat serum for 15 minutes. The sections were then incubated with a rabbit antibody against UCP1 (1 : 200, Abcam, Cambridge, UK) overnight at 4°C, which was followed by incubation with a biotinylated secondary antibody (Beyotime, China) for 30 minutes at room temperature and treatment with streptavidin-peroxidase complex (Beyotime, China) for 15 minutes. Staining was performed using diaminobenzidine (DAB). Finally, the sections were counterstained with hematoxylin and dehydrated in ethanol.

### 2.4. Real-Time Quantitative PCR

Total RNA was isolated from eWAT using RNAiso Plus (Takara, Dalian, China) according to the manufacturer's instructions, and cDNA was synthesized from RNA using the PrimeScript™ RT Master Mix (Takara). Primers for the analysis were designed as follows: *β*-actin, 5′-GTTCCGATGCCCTGAGGCTC and 5′-CAGACAGCACTGTGTTGGCA; UCP1, 5′-ACTGCCACACCTCCAGTCATT and 5′-CTTTGCCTCACTCAGGATTGG; PRDM16, 5′-CAGCACGGTGAAGCCATTC and 5′-GCGTGCATCCGCTTGTG; Cidea, 5′-TGCTCTTCTGTATCGCCCAGT and 5′-GCCGTGTTAAGGAATCTGCTG; PGC1a, 5′-CCCTGCCATTGTTAAGACC and 5′-TGCTGCTGTTCCTGTTTTC; HSL, 5′-TCCTGGAACTAAGTGGACGCAAG and 5′-CAGACACACTCCTGCGCATAGAC; ATGL, 5′-AACACCAGCATCCAGTTCAA and 5′-GGTTCAGTAGGCCATTCCTC; PPARa, 5′-CCCTGAACATCGAGTGTCGAA and 5′-TCGCCGAAAGAAGCCCTTA; PLIN1, 5′-GTCGTCATGGCTCTCATCCT and 5′-GGCCAACACTCTTTCTCGAC; PPAR?, 5′-ATTCTGGCCCACCAACTTCGG and 5′-TGGAAGCCTGATGCTTTATCCCCA; and AP2, 5′-TGGGAACCTGGAAGCTTGTCTC and 5′-GAATTCCACGCCCAGTTTGA. Quantitative real-time PCR was performed to analyze gene expression using the SYBR Premix Ex Tap II (Takara) and the LightCycler 480 System (Roche, Germany) (*n* = 6–8 mice per group). The mRNA levels were normalized to *β*-actin.

### 2.5. Western Blot Analysis

Proteins were extracted from eWAT using a lysis buffer containing protease and phosphatase inhibitors (KeyGen Biotech, Nanjing, China) by homogenization and were then centrifuged at 13000*g* for 10 minutes at 4°C (*n* = 3 mice per group). The protein concentration was determined using a BCA kit (Beyotime Biotechnology, China). The protein extracts were boiled for 8 minutes at 100°C and then stored at −20°C. After 12% SDS-acrylamide gel electrophoresis, the proteins were transferred onto polyvinylidene fluoride (PVDF) membranes (Millipore, USA) and were then blocked using 5% nonfat dry milk in Tris-buffered saline with 0.05% Tween 20 for 2 h. The membranes were incubated with primary antibody dilutions overnight at 4°C, including a mouse antibody against *β*-actin (1 : 2000, Zhongshan Golden Bridge, China), rabbit antibody against UCP1 (1 : 1000, Abcam, Cambridge, UK), rabbit antibody against phospho-p38 MAPK (Thr180/Tyr182) (1 : 1000, Cell Signaling Technology, USA), rabbit antibody against PKA (1 : 1000, Cell Signaling Technology), rabbit antibody against phospho-PKA (Thr197) (1 : 1000, Cell Signaling Technology), rabbit antibody against HSL (1 : 1000, Cell Signaling Technology), and rabbit antibody against PGC1a (1 : 1000, Wanleibio, China). The membranes were washed three times using Tris-buffered saline with 0.05% Tween 20 and were incubated for 2 h with horseradish peroxidase- (HRP-) labeled anti-rabbit or anti-mouse IgG (1 : 5000, Zhongshan Golden Bridge, China). Antibody-specific expression was detected using the DNR MicroChemi System (DNR Bio-Imaging Systems Ltd., USA) and was analyzed using ImageJ software.

### 2.6. Statistical Analysis

All data are presented as the means ± SE. The statistical analyses were performed using one-way analysis of variance (ANOVA) and SPSS 22.0 software (SPSS Inc., Chicago, IL, USA), followed by the least significant difference (LSD) test for multiple comparisons. *P* < 0.05 was considered statistically significant.

## 3. Results

### 3.1. PYC Reduced Body Weight Gain in ApoE-Deficient Mice

**Figure 1:**
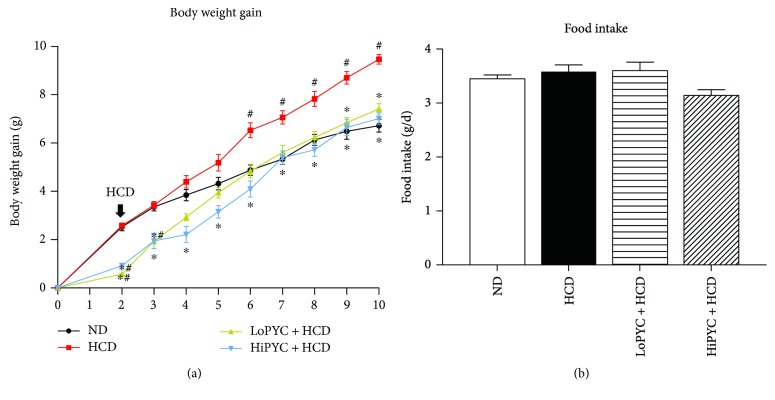
PYC lowered body weight gain. (a) Change in body weight gain and (b) daily food intake. The data are expressed as the means ± SE for 20 to 25 mice per group. ^#^*p* < 0.05 versus the ND group; ^∗^*p* < 0.05 versus the HCD group.

To investigate the preventative effect of PYC on obesity, mice received the PYC treatment for 10 weeks. After 2 weeks, the body weight gain was 0.76, 0.92, and 2.55 g for the LoPYC + HCD, HiPYC + HCD, and blank groups, respectively (Figure 1(a)). The significant difference between the PYC-treated and blank groups suggested that PYC may have a preventative effect on obesity. The blank group was divided into the ND group and the HCD group. The mice in the HCD and PYC-treated groups were given an HCD for 8 weeks, and body weight was measured weekly. Body weight gain occurred faster in the HCD group than in the ND group. This increase differed significantly between the HCD and ND groups starting at 6 weeks. The PYC decreased the HCD-induced body weight gain throughout the experiment, although there were significant differences only between the LoPYC + HCD group and the HCD group starting at 9 weeks (Figure 1(a)). The data confirmed the preventative effect of PYC on obesity. However, there was no difference in food intake between the HCD and PYC-treated groups (Figure 1(b)).

### 3.2. PYC Reduced Visceral Fat and the Size of Adipocytes within eWAT

**Figure 2:**
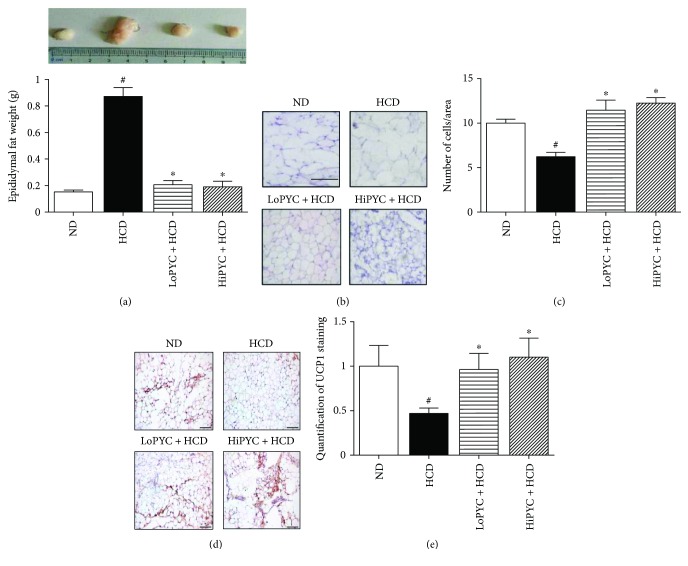
PYC decreased the eWAT mass and the size of adipocytes within eWAT. (a) The eWAT mass; (b) H&E staining of eWAT to observe the size of adipocytes, scale bar = 50 *μ*m; and (c) the number of cells/area in H&E-stained sections. (d) UCP1 staining in eWAT. Pictures are shown at 20x magnification; scale bar = 100 *μ*m. (e) Quantification of UCP1 staining in eWAT. The data are expressed as the means ± SE for 5 mice per group. ^#^*p* < 0.05 versus the ND group; ^∗^*p* < 0.05 versus the HCD group.

The HCD-induced excessive fat accumulation was assessed by measuring the eWAT mass. The eWAT mass in the HCD mice was greater than that in the ND mice (0.87 ± 0.06 g versus 0.15 ± 0.01 g). The eWAT mass in the low-dose and high-dose PYC-treated mice was significantly lower than that in the HCD mice (0.21 ± 0.03 g, 0.19 ± 0.04 g versus 0.87 ± 0.06 g) (Figure 2(a)). The H&E staining revealed that PYC supplementation significantly decreased the size of adipocytes in eWAT (Figure 2(b)). The number of cells/area in the H&E-stained sections was counted, and the results suggested that the number of cells in the PYC-treated mice was significantly greater than that in the HCD mice (Figure 2(c)).

### 3.3. PYC Altered the Expression of Genes Related to Lipid Metabolism

**Figure 3:**
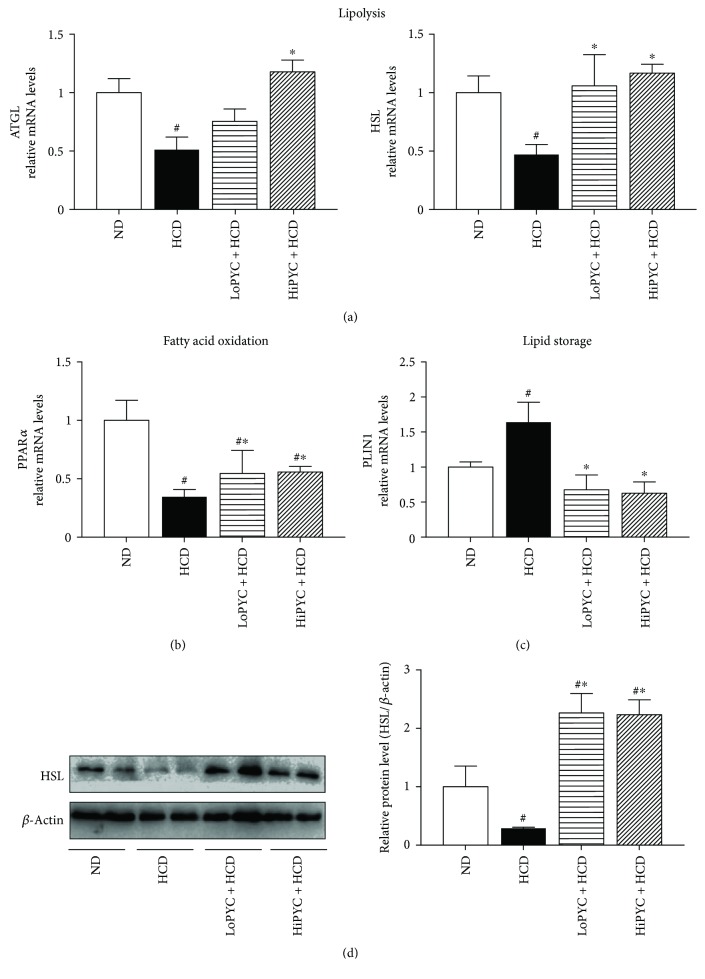
PYC altered the expression of genes related to lipid metabolism. (a) The mRNA levels of genes (ATGL and HSL) related to lipolysis; (b) the mRNA level of PPAR*α* (related to fatty acid oxidation); (c) the mRNA level of PLIN1; and (d) the protein level of HSL. The data are expressed as the means ± SE for 6 to 8 mice per group. ^#^*p* < 0.05 versus the ND group; ^∗^*p* < 0.05 versus the HCD group.

To determine the likely mechanism by which PYC decreased the mass of eWAT, we assessed the expression of genes related to lipid metabolism. The expression of ATGL and HSL, genes that are related to lipolysis, was significantly increased in the PYC-treated groups compared with that in the HCD group. The expression of HSL mRNA was more than 2-fold greater in the PYC-treated groups than in the HCD group. Consistent with these results, the expression of ATGL mRNA in the HiPYC + HCD group was 2-fold higher than that in the HCD group, although this increase was not significantly different between the LoPYC + HCD group and the HCD group (Figure 3(a)). In addition, the results of Western blot analysis showed that the protein level of HSL in the PYC-treated groups was higher than that in the HCD group (Figure 3(d)). Moreover, PPAR*α* has been shown to play a role in regulating fatty acid oxidation [21]. The mRNA level of PPAR*α* in the low-dose and high-dose PYC-treated mice was 2-fold higher than that in the HCD mice (Figure 3(b)). PLIN1, a member of the PAT family, is a key gene that regulates lipid storage in WAT [22–24]. The mRNA level of PLIN1 in the HCD mice was 1.6-fold greater than that in the ND mice, while the levels in the low-dose and high-dose PYC-treated mice were approximately 58% and 62% lower than those in the HCD mice, respectively (Figure 3(c)).

### 3.4. PYC Enhanced the Expression of Genes Related to WAT Browning.

**Figure 4:**
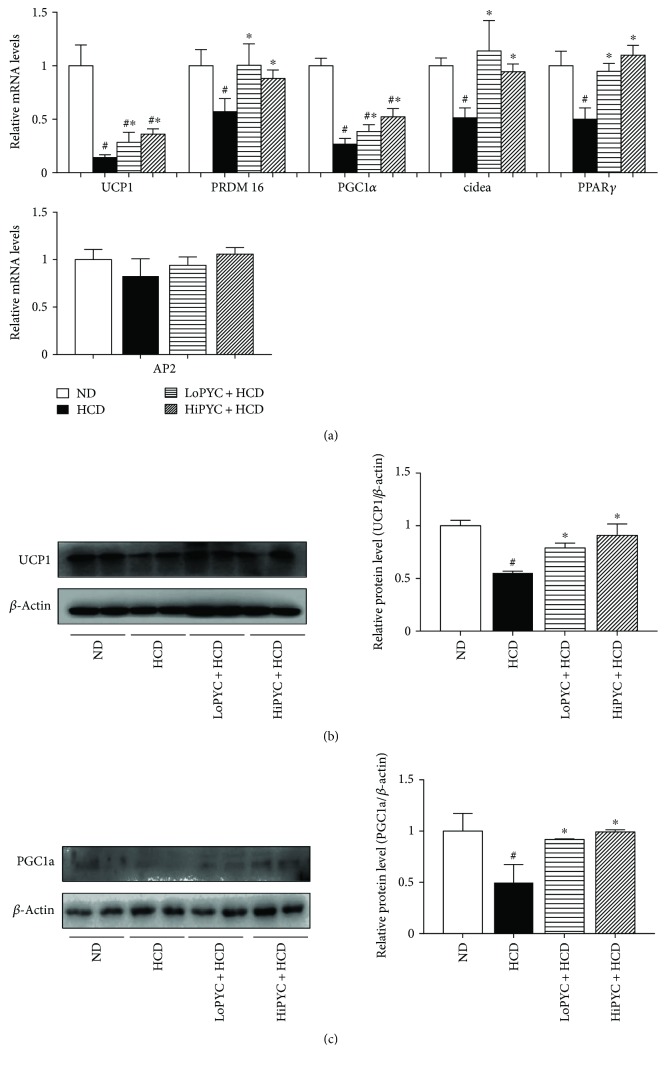
PYC enhanced the expression of genes and proteins related to WAT browning. (a) The expression of genes related to WAT browning and the protein levels of (b) UCP1 and (c) PGC1*α*. The data are expressed as the means ± SE for 6 to 8 mice per group. ^#^*p* < 0.05 versus the ND group; ^∗^*p* < 0.05 versus the HCD group.

The increase in brown fat-specific proteins, such as UCP1, PRDM16, Cidea, and PGC1*α*, in white adipocytes induces its acquisition of brown adipocyte features [12, 13]. To investigate the effect of PYC on WAT browning, we analyzed brown fat-specific gene expression in eWAT. The mRNA level of UCP1 in the HCD-fed mice was 80% less than that in the ND-fed mice (Figure 4(a)). The level of UCP1 in the LoPYC + HCD group was 2-fold higher than that in the HCD group, while that in the HiPYC + HCD group was 2.5-fold greater (Figure 4(a)). Consistent with these results, the mRNA levels of genes related to WAT browning, such as PRDM16, PGC1*α*, Cidea, and PPAR*γ*, showed a similar trend (Figure 4(a)). The mRNA levels of PRDM16, PGC1*α*, Cidea, and PPAR*γ* in the HCD-fed mice were approximately 45%, 73%, 50%, and 50% lower than those in the ND-fed mice, respectively. The low and high PYC doses reversed the phenomenon that resulted from the HCD. The level of PRDM16 was increased approximately 1.75- and 1.5-fold in the LoPYC + HCD and HiPYC + HCD mice, respectively, compared with that in the HCD-fed mice. The level of PGC1*α* was increased approximately 1.7-fold in the LoPYC + HCD mice compared with that in the HCD-fed mice, while there was nearly a 2-fold increase in the HiPYC + HCD group compared with the HCD group. Cidea and PPAR*γ* mRNA levels exhibited an approximately 2-fold increase in the LoPYC + HCD and HiPYC + HCD mice compared with those in the HCD-fed mice. However, PYC did not affect the expression of AP2 (Figure 4(a)). PYC increased the number of UCP1-positive cells (Figures 2(d) and 2(e)). In addition, the protein levels of UCP1 and PGC1 in eWAT from the PYC-treated groups were higher than those in the HCD group (Figures 4(b) and 4(c)).

### 3.5. PYC Increased the Expression of Proteins Related to the PKA Signaling Pathway

**Figure 5:**
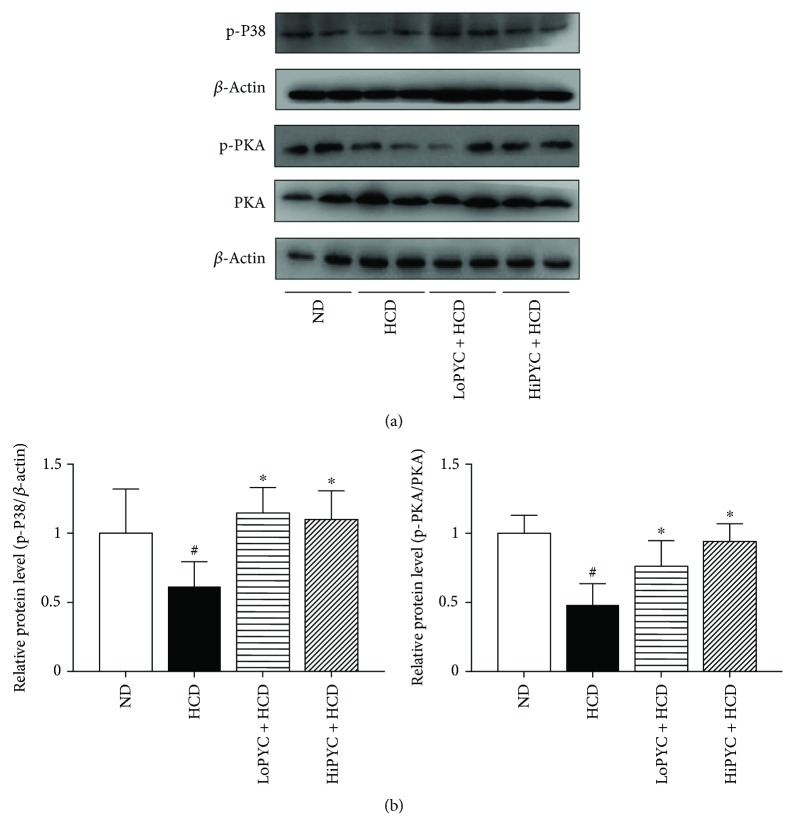
PYC increased the expression of proteins related to the PKA signaling pathway. (a) Results of the Western blot analysis of PKA signaling in eWAT, including p-p38 MAPK, p-PKA, and PKA, and (b) quantification of the bands. The data are expressed as the means ± SE for 3 mice per group. ^#^*p* < 0.05 versus the ND group; ^∗^*p* < 0.05 versus the HCD group.

PKA signaling activates UCP1 expression, which has a protective effect on metabolic health [25]. To assess whether PYC could enhance the expression of UCP1 through the PKA-dependent pathway, we detected the expression of proteins related to the PKA signaling pathway. As expected, PYC significantly increased the phosphorylation of PKA and p38 proteins in eWAT (Figure 5).

## 4. Discussion

Obesity is a common chronic disease that results from an imbalance of food intake and energy expenditure. Generally, classic brown adipocytes and beige adipocytes increase energy expenditure by generating heat, which protects against the development of overweight and obesity. In our study, we investigated for the first time the effect of PYC on the browning of WAT by altering gene expression related to beige adipogenesis in ApoE-deficient mice, which provides new insight into the effect of PYC on obesity.

In our study, PYC supplementation reduced body weight gain compared with the HCD group, which is consistent with previous in vivo studies [11, 26, 27]. The morphological results showed that the PYC-treated mice appeared to have smaller-sized adipocytes within eWAT than the obese mice, suggesting that PYC may improve the lipid accumulation within eWAT caused by HCD. It is recognized that obesity is characterized by abnormal fat deposition, suggesting that it is necessary to investigate changes in genes or proteins in adipose tissue. To explore the likely mechanism of the obesity-preventative effect of PYC, we analyzed genes and proteins related to the browning of WAT and lipid metabolism.

The browning of WAT provides a new perspective in the identification of therapeutic strategies for weight loss. Several studies have focused on the relationship between the antiobesity effects of plant extracts and WAT browning [28–31]. In our study, we found that PYC significantly increased the expression of UCP1, including the mRNA and protein levels. UCP1, a thermogenic protein, is abundantly expressed in BAT. The increase in UCP1 expression in WAT induces the formation of beige adipocytes [12, 13]. The UCP1-positive beige adipose tissue also has a thermogenic capability that is similar to that of BAT [9, 32, 33]. PYC increased the UCP1 expression in eWAT, indicating that PYC possesses the ability to promote WAT browning, thereby increasing energy expenditure and thermogenesis. A recent study showed that UCP1 activity is stimulated by the cAMP-dependent PKA pathway. The activation of PKA triggers p38 MAPK phosphorylation, which phosphorylates PGC1*α* [10, 25, 34]. PGC1*α* is a transcriptional coactivator that modulates the expression of UCP1 and then stimulates thermogenesis and the browning of WAT [35–37]. The present study showed that PYC significantly increased PKA phosphorylation and that the protein expression of p-p38 MAPK and PGC1*α* was higher in the PYC-treated groups than in the HCD group. The findings suggested that the increased expression of UCP1 was regulated by the PKA-p38 MAPK-PGC1*α* signaling pathway by PYC in ApoE-deficient mice fed with an HCD. Furthermore, the expression levels of other brown fat-specific markers, such as PRDM16, Cidea, and PPAR*γ*, were increased in the PYC-treated mice, which explains the effect of PYC on WAT browning.

Lipid metabolism is a complex process that involves multiple genes. Among them, ATGL and HSL both hydrolyze triglycerides (TGs) within lipid droplets, leading to the release of diacylglycerol (DG) and fatty acids [38, 39]. HSL is capable of simultaneously hydrolyzing DG [40]. This process is called lipolysis, and a portion of the fatty acids produced in the process is oxidized to provide energy. PYC supplementation increased the levels of ATGL and HSL in adipose tissue; however, PKA inhibitor (H89) treatment clearly blocked the level of HSL, suggesting that PYC promotes lipolysis via a PKA-dependent pathway [11, 41]. The activation of PKA further induces downstream HSL phosphorylation. As a result, a large number of fatty acids are released, and free fatty acids also stimulate UCP1 activity [42–45]. Consistent with these findings, we also found that ATGL and HSL levels were upregulated in the PYC-treated mice, which may explain why there was less lipid droplet accumulation in the PYC-treated groups than in the obese mice. Additionally, HCD significantly increased the expression of PLIN1, while PYC treatment clearly decreased PLIN1 expression. PLIN1, a member of the PAT family, is located on the surface of lipid droplets and plays a role in preventing lipolysis under basal conditions [44, 46]. PYC also promoted lipolysis by reducing the expression of PLIN1. These results also revealed that PYC may promote lipolysis through the PKA signaling pathway and that lipolysis induced by PYC promoted the expression of UCP1. Furthermore, we found that PYC could reverse the reduction in PPAR*α* expression resulting from an HCD. PPAR*α* is a nuclear receptor that plays an important role in the transcriptional control of genes encoding fatty acid *β*-oxidation enzymes [21, 47]. It has been reported that a PPAR*α* agonist induced beige cell formation [48]. Together with the results of our study, these results suggest that PYC may promote WAT browning by upregulating PPAR*α* expression.

## 5. Conclusion

**Figure 6:**
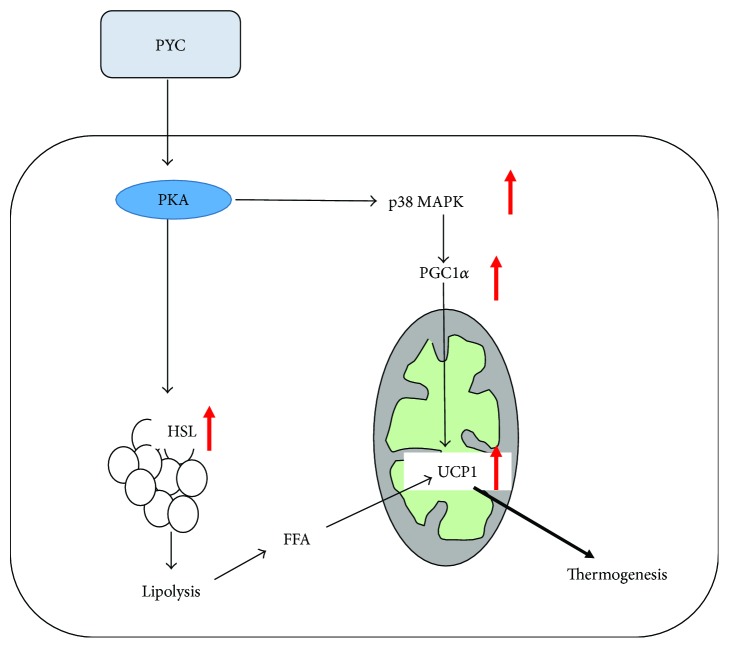
The likely mechanism underlying the effect of PYC on the browning of WAT. PYC may stimulate UCP1 expression through the PKA-p38 MAPK-PGC1*α* pathway and the PKA-HSL pathway.

In conclusion, our data demonstrate that PYC can effectively reduce body weight and body fat deposition. Regarding the underlying mechanism, PYC promotes lipolysis and WAT browning via the PKA signaling pathway in eWAT (Figure 6). This finding suggests that PYC administration may be a novel strategy for preventing and treating obesity and associated metabolic diseases.
